# Supplementary material for: Single-cell analysis of human MAIT cell transcriptional, functional and clonal diversity
Source: Nat Immunol. 2023 Aug 14;24(9):1565–78. doi: 10.1038/s41590-023-01575-1 (PMC10457204; doi:10.1038/s41590-023-01575-1)
Supplement: Supplementary file 1 — Supplementary Figs. 1–3. [file 41590_2023_1575_MOESM1_ESM.pdf]

nature immunology

Resource

https://doi.org/10.1038/s41590-023-01575-1

Single-cell analysis of human MAIT cell transcriptional, functional and clonal diversity

In the format provided by the authors and unedited

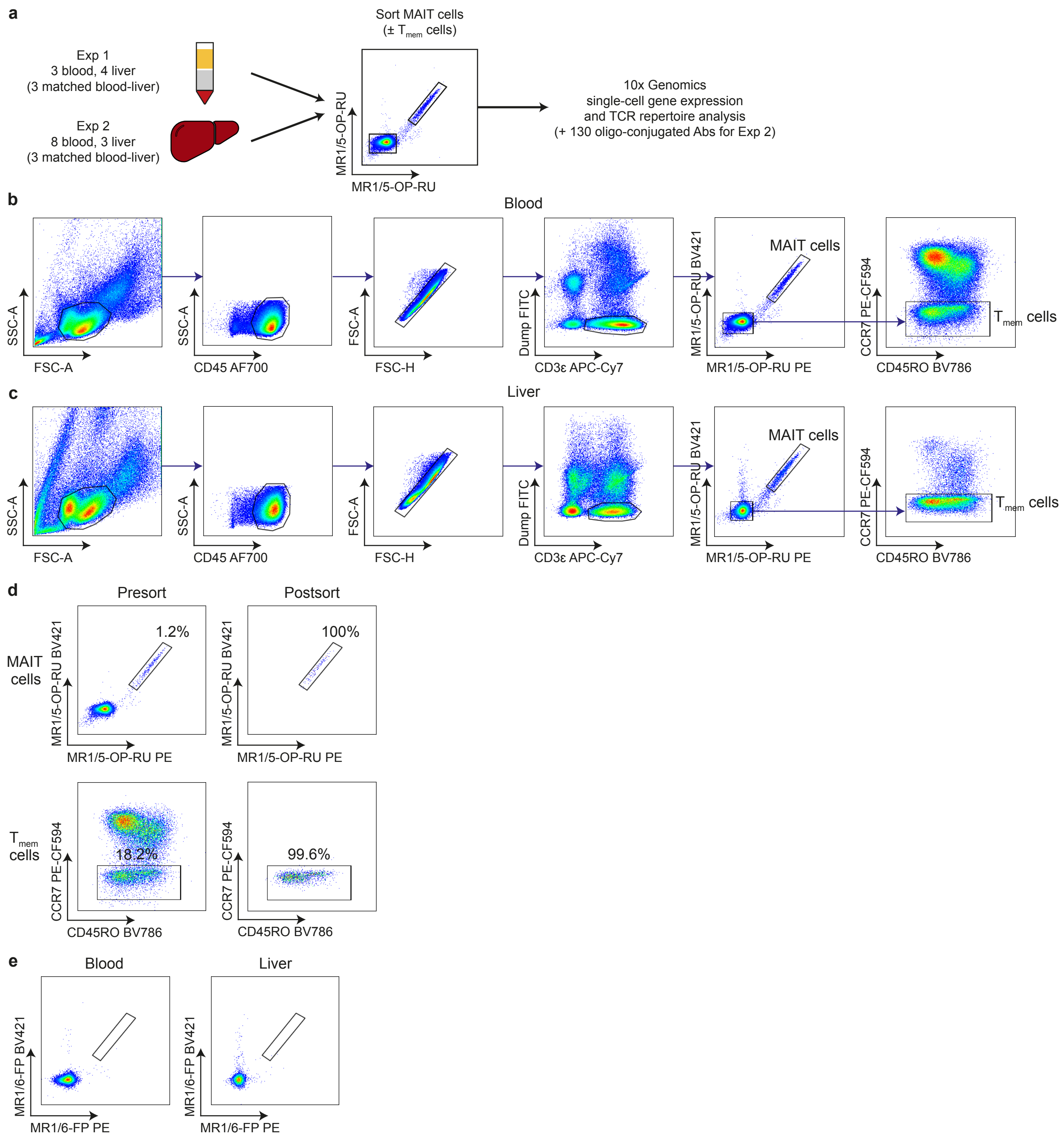

Supplementary Fig. 1 | Blood-liver experiments schematic and gating strategy. a, Schematic illustrating the experimental protocol for Exp 1 and Exp 2. **b**,**c**, Gating strategy for sorting MAIT cells (CD3$^+$MR1/5-OP-RU$^+$) and conventional memory T (T_{mem}) cells (CD3$^+$MR1/5-OP-RU$^-$CCR7$^-$) from matched human blood (**b**) and liver (**c**). Dump channel contained antibodies to CD14, CD19, TCR γδ, TCR Vα24-Jα18, and TCR Vδ2, plus SYTOX Green Nucleic Acid Stain. **d**, FACS plots for a representative sample showing MAIT and T_{mem} cells presort and postsort. Sort purity was > 99%. **e**, FACS plots for representative blood and liver samples stained with the negative control MR1/6-FP tetramer.

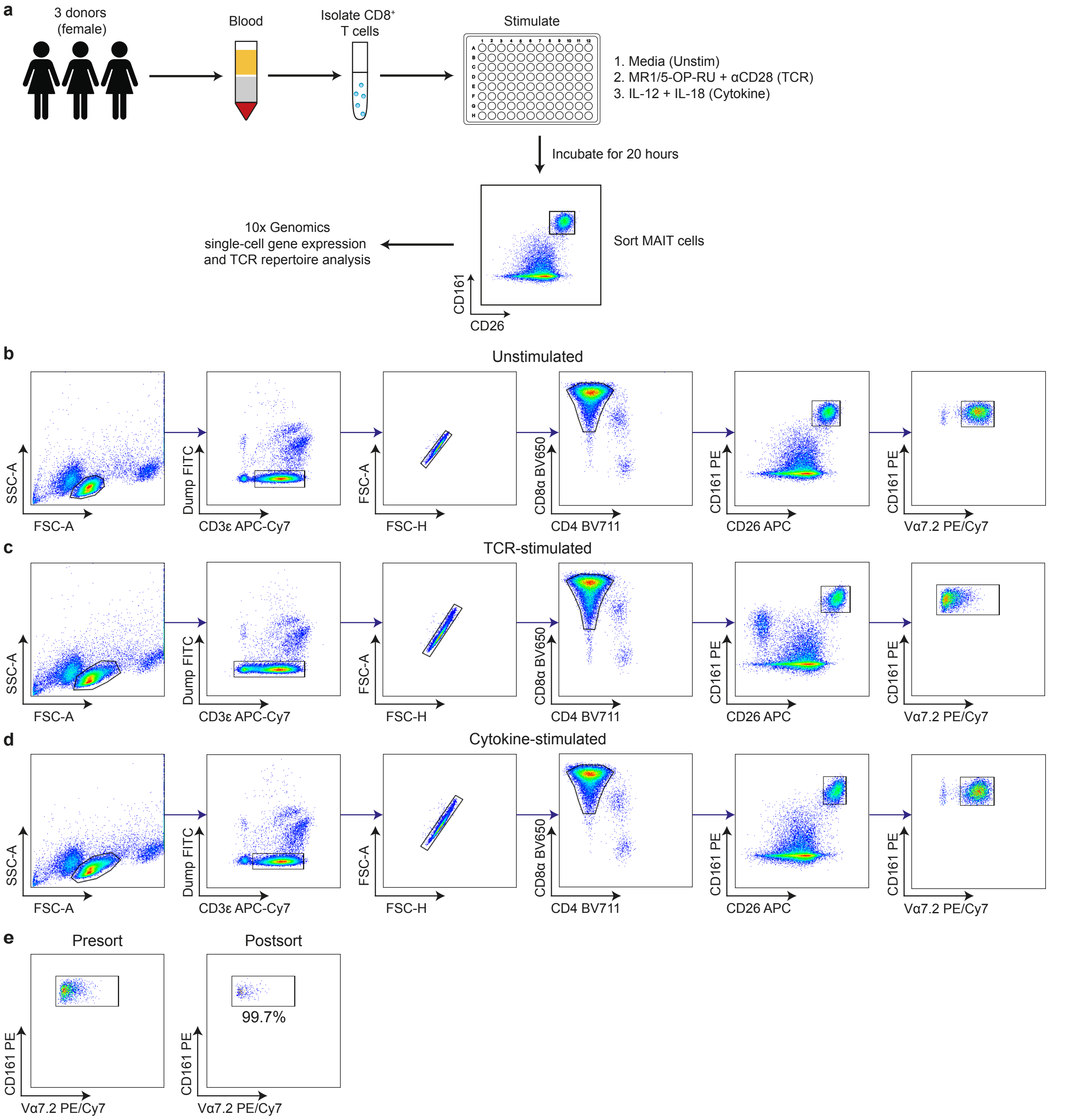

Supplementary Fig. 2 | Exp 3 schematic and gating strategy. a, Schematic illustrating the experimental protocol for Exp 3. **b**-**d**, Gating strategy for sorting MAIT cells ($CD3^{+}CD8^{+}CD26^{+}CD161^{hi}V\alpha7.2^{+}$) from unstimulated (**b**), TCR-stimulated (**c**), and cytokine-stimulated (**d**) isolated $CD8^{+}$ cells. Due to TCR downregulation within the TCR stimulation condition (**c**), MAIT cells were sorted as $CD8^{+}CD26^{+}CD161^{hi}$ lymphocytes. The $CD26^{-}CD161^{int}$ lymphocytes are $CD3^{-}CD8^{low}$ and are likely to be NK cells. Dump channel contained antibodies to CD14, CD19, TCR γδ, TCR Vα24-Jα18, and TCR Vδ2, plus SYTOX Green Nucleic Acid Stain. **e**, FACS plots for a representative sample (TCR stimulation condition) showing MAIT cells presort and postsort. Sort purity was > 99%.

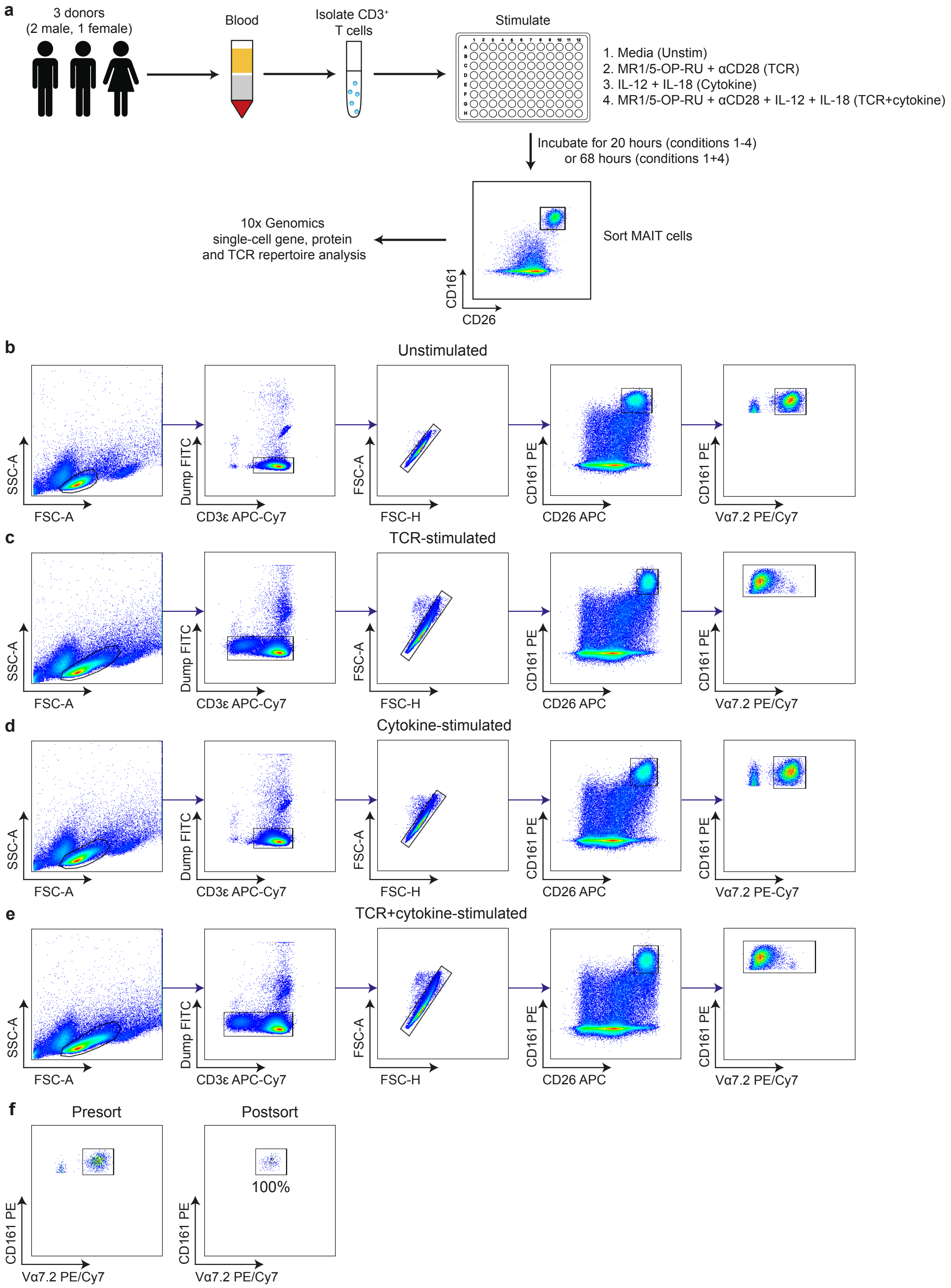

Supplementary Fig. 3 | Exp 4 schematic and gating strategy. a, Schematic illustrating the experimental protocol for Exp 4. **b**-**e**, Gating strategy for sorting MAIT cells ($CD3^+CD26^+CD161^{hi}V\alpha7.2^+$) from unstimulated (**b**), TCR-stimulated (**c**), cytokine-stimulated (**d**), and TCR+cytokine-stimulated (**e**) isolated $CD3^+$ cells. Due to TCR downregulation within the TCR and TCR+cytokine stimulation conditions (**c**, **e**), MAIT cells were sorted as $CD26^+CD161^{hi}$ lymphocytes. Dump channel contained antibodies to CD14, CD19, TCR γδ, TCR Vα24-Jα18, and TCR Vδ2, plus SYTOX Green Nucleic Acid Stain. **f**, FACS plots for a representative sample (unstimulated condition) showing MAIT cells presort and postsort. Sort purity was > 99%.
