# Supplementary material for: Single-cell analysis of human MAIT cell transcriptional, functional and clonal diversity
Source: Nat Immunol. 2023 Aug 14;24(9):1565–78. doi: 10.1038/s41590-023-01575-1 (PMC10457204; doi:10.1038/s41590-023-01575-1)
Supplement: Supplementary file 2 — Reporting Summary [file 41590_2023_1575_MOESM2_ESM.pdf]

Corresponding author(s): Paul Klenerman, Lucy C. Garner

Last updated by author(s): Jul 30, 2023

Reporting Summary

Nature Portfolio wishes to improve the reproducibility of the work that we publish. This form provides structure for consistency and transparency in reporting. For further information on Nature Portfolio policies, see our Editorial Policies and the Editorial Policy Checklist.

Statistics

For all statistical analyses, confirm that the following items are present in the figure legend, table legend, main text, or Methods section.

n/a	Confirmed	
☐	☒	The exact sample size (*n*) for each experimental group/condition, given as a discrete number and unit of measurement
☐	☒	A statement on whether measurements were taken from distinct samples or whether the same sample was measured repeatedly
☐	☒	The statistical test(s) used AND whether they are one- or two-sided<br>*Only common tests should be described solely by name; describe more complex techniques in the Methods section.*
☐	☒	A description of all covariates tested
☐	☒	A description of any assumptions or corrections, such as tests of normality and adjustment for multiple comparisons
☐	☒	A full description of the statistical parameters including central tendency (e.g. means) or other basic estimates (e.g. regression coefficient) AND variation (e.g. standard deviation) or associated estimates of uncertainty (e.g. confidence intervals)
☐	☒	For null hypothesis testing, the test statistic (e.g. *F*, *t*, *r*) with confidence intervals, effect sizes, degrees of freedom and *P* value noted<br>*Give P values as exact values whenever suitable.*
☒	☐	For Bayesian analysis, information on the choice of priors and Markov chain Monte Carlo settings
☒	☐	For hierarchical and complex designs, identification of the appropriate level for tests and full reporting of outcomes
☐	☒	Estimates of effect sizes (e.g. Cohen's *d*, Pearson's *r*), indicating how they were calculated

Our web collection on statistics for biologists contains articles on many of the points above.

Software and code

Policy information about availability of computer code

Data collection

Flow cytometry data were collected using BD FACSDiva Software (v8.0.1). Sequencing data were collected using HiSeq Control Software (v2.2.68; Illumina) or NovaSeq Control Software (v1.6.0-1.8.0; Illumina).

Data analysis

Flow cytometry data were analysed using FlowJo (v10.8.1; BD Biosciences).

R version 4.1.1-4.2.0. R packages: AUCell (v1.16.0-1.20.1), biomaRt (v2.54.1), cellhashR (v1.0.3), clusterProfiler (v4.7.1), clustree (v0.4.3-0.5.0), ComplexHeatmap (v2.14.0), DropletQC (v0.0.0.9000), edgeR (v3.24.3), EMT (v1.3), ggplot2 (v3.3.4-3.4.2), ggpubr (v0.4.0), ggrepel (v0.9.1-0.9.3), ggseqlogo (v0.1), gridExtra (v2.3), Harmony (v0.1.1), MAST (v1.18.0-1.24.1), org.Hs.eg.db (v3.13.0-3.16.0), pheatmap (v1.0.12), plotrix (v3.8.2), ReactomePA (v1.36.0), readxl (v1.4.2), rstatix (v0.7.2), scales (v1.1.1-1.2.1), scater (v1.20.1-1.26.1), sctransform (v0.3.2-0.3.5), SCORPIUS (v1.0.8), Seurat (v4.0.3-4.3.0), Slingshot (v2.0.0), STACAS (v2.0.1), tidyverse (v1.3.1-2.0.0), vegan (v2.6.4), venn (v1.11), VennDiagram (v1.7.3), viridis (v0.6.1-0.6.2).

Python version 3.7.10-3.10.8. Python packages: pandas (v1.2.5-1.5.2), pySCENIC (v0.11.2-0.12.1).

Command-line software: bedtools (v2.26.0), Bowtie 2 (v2.3.4.1), Cell Ranger (v3.0.1-7.0.1; 10x Genomics), deepTools (v3.1.0-3.5.1), FastQC (v0.11.5), featureCounts (v1.6.0), HOMER (v4.8), MACS2 (v2.1.1), Picard (v2.15.0), samtools (v1.6), Trimmomatic (v0.36).

Other software: GraphPad Prism (v9.5.1; GraphPad Software, LLC), IMGT/JunctionAnalysis (v2.3.0; https://www.imgt.org/IMGT_jcta/analysis), GSEA (v4.3.2), Integrative Genomics Viewer (v2.16.0).

For manuscripts utilizing custom algorithms or software that are central to the research but not yet described in published literature, software must be made available to editors and reviewers. We strongly encourage code deposition in a community repository (e.g. GitHub). See the Nature Portfolio guidelines for submitting code & software for further information.

Data

Policy information about availability of data

All manuscripts must include a data availability statement. This statement should provide the following information, where applicable:

- Accession codes, unique identifiers, or web links for publicly available datasets
- A description of any restrictions on data availability
- For clinical datasets or third party data, please ensure that the statement adheres to our policy

Sequencing data generated in this study have been deposited in NCBI's Gene Expression Omnibus (GEO) and are accessible through GEO SuperSeries accession number GSE194189. The Bioconductor org.Hs.eg.db annotation package and ENSEMBL_MART_ENSEMBL BioMart database are publicly available.

Research involving human participants, their data, or biological material

Policy information about studies with human participants or human data. See also policy information about sex, gender (identity/presentation), and sexual orientation and race, ethnicity and racism.

Reporting on sex and gender	Blood-liver scRNA-seq experiments: the sex of liver patients is listed in Supplementary Table 1. Blood was additionally obtained from one male and four females. Stimulation scRNA-seq experiments: for Exp 3, blood was obtained from three females; for Exp 4, blood was obtained from two males and one female. Experiments were underpowered for formal sex-based analysis. Sex information is not available for the donors used in flow cytometry validation experiments.
Reporting on race, ethnicity, or other socially relevant groupings	Race and ethnicity information is not available for the donors in this study.
Population characteristics	Liver patient information, including age, sex, diagnosis and treatment is detailed in Supplementary Table 1. Age and gender were not accounted for in the analyses. Donor was included as a covariate for differential expression analyses between conditions e.g. cell types, tissues, stimuli.
Recruitment	Human participants were recruited at the Churchill Hospital, Oxford, UK and the University Hospital Basel, Basel, Switzerland. Patients had no chronic liver disease, active excess alcohol consumption (> 14 g/day), infection, immunosuppression or family history of liver disease. The requirement for written informed consent may introduce selection bias. Patient recruitment was influenced by practical considerations, such as the time of day of the surgery and the amount of liver tissue available.
Ethics oversight	Samples were obtained with written informed consent through the Oxford Gastrointestinal Illnesses Biobank (REC ref. 16/YH/0247) or under Ethikkommission Nordwest- und Zentralschweiz (EKNZ) numbers EKNZ-2014-362, EKNZ-2016-01188 and EKNZ-2019-02118.

Note that full information on the approval of the study protocol must also be provided in the manuscript.

Field-specific reporting

Please select the one below that is the best fit for your research. If you are not sure, read the appropriate sections before making your selection.

☒ Life sciences ☐ Behavioural & social sciences ☐ Ecological, evolutionary & environmental sciences

For a reference copy of the document with all sections, see nature.com/documents/nr-reporting-summary-flat.pdf

Life sciences study design

All studies must disclose on these points even when the disclosure is negative.

Sample size	Statistical methods were not used to predetermine sample size. scRNA-seq and flow cytometry experiments included ≥ 3 donors per group (tissue or stimulation condition). Initial blood-liver (Exp 1) and stimulation (Exp 3) scRNA-seq experiments were followed by validation experiments (Exp 2 and 4) – the findings of these were highly concordant.
Data exclusions	No samples were excluded from scRNA-seq, ATAC-seq or flow cytometry datasets.<br><br>scRNA-seq cell exclusions: cells with low unique molecular identifier counts, low gene counts and/or a high percentage of mitochondrial reads were assumed to be dead/dying cells and were removed. For Exp 1 and 2, cells labelled as empty droplets or damaged cells by DropletQC were removed (damaged cells in Exp 4 also removed). For Exp 2 and 4, only cells called as consensus singlets by hashtag demultiplexing (cellhashR) were retained. Cells with two TCRα and two TCRβ chains, or more than two TCRα and/or TCRβ chains, were assumed to be doublets and discarded.
Replication	Initial blood-liver (Exp 1) and stimulation (Exp 3) scRNA-seq experiments were followed by validation experiments (Exp 2 and 4) – the findings of these were highly concordant. Genes of interest from Exp 1 and 3 were validated at the protein level by CITE-seq (Exp 2 and 4) and flow cytometry. As described in the methods, pySCENIC transcription factor regulon analysis was performed 100 times and results aggregated to define high-confidence regulons.

	ATAC-seq was performed once with three donors.<br><br>Activation markers and cytokines identified by scRNA-seq were validated by flow cytometry in 14 donors (three independent experiments) and 11 donors (two independent experiments), respectively. Stimulation of sorted CD56- and CD56+ MAIT cells was performed once with three donors. All attempts at replication were successful.
Randomization	For blood-liver scRNA-seq experiments, randomization to groups was not possible since liver patients were compared with healthy donors. For stimulation experiments, cells from each donor were present in all conditions.<br><br>Blood-liver scRNA-seq experiments (Exp 1 and 2) were each performed in two batches - donors were randomly distributed across batches. Stimulation scRNA-seq experiments (Exp 3 and 4) were each performed as a single batch. Donor was included as a covariate in differential expression analyses.
Blinding	Experiments and analyses were not performed blinded as the same investigator performed sample collection, sample processing, data generation and data analysis.

Reporting for specific materials, systems and methods

We require information from authors about some types of materials, experimental systems and methods used in many studies. Here, indicate whether each material, system or method listed is relevant to your study. If you are not sure if a list item applies to your research, read the appropriate section before selecting a response.

Materials & experimental systems

n/a	Involved in the study
☐	☒ Antibodies
☒	☐ Eukaryotic cell lines
☒	☐ Palaeontology and archaeology
☒	☐ Animals and other organisms
☒	☐ Clinical data
☒	☐ Dual use research of concern
☒	☐ Plants

Methods

n/a	Involved in the study
☒	☐ ChIP-seq
☐	☒ Flow cytometry
☒	☐ MRI-based neuroimaging

Antibodies

Antibodies used

Specificity; fluorophore; clone; manufacturer; catalogue number; RRID

Anti-human CCL4; APC; REA511; Miltenyi Biotec; 130-129-199; AB_2922004
Anti-human CCR7; PE-CF594; 150503; BD Biosciences; 562381; AB_11153301
Anti-human CD3ε; APC-Cy7; HIT3A; BioLegend; 300318; AB_314054
Anti-human CD3ε; APC-Cy7; UCHT1; BioLegend; 300426; AB_830755
Anti-human CD3ε; BV650; UCHT1; BioLegend; 300468; AB_2629574
Anti-human CD3ε; BV650; UCHT1; BioLegend; 300464; AB_2566036
Anti-human CD3ε; FITC; UCHT1; BioLegend; 300406; AB_314060
Anti-human CD3ε; PerCP-Cy5.5; UCHT1; BioLegend; 300430; AB_893299
Anti-human CD4; BV650; OKT4; BioLegend; 317436; AB_2563050
Anti-human CD4; BV711; OKT4; BioLegend; 317440; AB_2562912
Anti-human CD4; BV785; OKT4; BioLegend; 317442; AB_2563242
Anti-human CD8α; AF700; SK1; BioLegend; 344724; AB_2562790
Anti-human CD8α; BV650; SK1; BioLegend; 344730; AB_2564510
Anti-human CD14; APC-Cy7; M5E2; BioLegend; 301820; AB_493695
Anti-human CD14; FITC; M5E2; BioLegend; 301804; AB_314186
Anti-human CD19; APC-Cy7; HIB19; BioLegend; 302218; AB_314248
Anti-human CD19; FITC; HIB19; BioLegend; 302206; AB_314236
Anti-human CD25; PerCP-Cy5.5; BC96; BioLegend; 302626; AB_2125478
Anti-human CD26; APC; BA5b; BioLegend; 30271; AB_10916120
Anti-human CD45; AF700; HI30; BioLegend; 304024; AB_493761
Anti-human CD45RO; BV786; UCHL1; BD Biosciences; 564290; AB_2738733
Anti-human CD56; BV421; HCD56; BioLegend; 318328; AB_11218798
Anti-human CD69; PerCP-Cy5.5; FN50; BioLegend; 310926; AB_2074956
Anti-human CD94; FITC; DX22; BioLegend; 305504; AB_314534
Anti-human CD137 (4-1BB); BV421; 4B4-1; BD Biosciences; 564091; AB_2722503
Anti-human CD154 (CD40L); BV785; 24-31; BioLegend; 310842; AB_2572187
Anti-human CD161; APC; 191B8; Miltenyi Biotec; 130-113-590; AB_2733346
Anti-human CD161; PE; 191B8; Miltenyi Biotec; 130-113-592; AB_2733625
Anti-human CD161; PE-Vio770; 191B8; Miltenyi Biotec; 130-113-594; AB_2751134
Anti-human Granzyme B; AF647; GB11; BioLegend; 515406; AB_2566333
Anti-human Granzyme B; Pacific Blue; GB11; BioLegend; 515408; AB_2562196
Anti-human IFNγ; BV605; 4S.B3; BioLegend; 502536; AB_2563881
Anti-human IFNγ; BV711; 4S.B3; BioLegend; 502540; AB_2563506

Anti-human Perforin; BV711; dG9; BioLegend; 308130; AB_2687190
Anti-human TCR γδ; BV480; B1; BD Biosciences; 566076; AB_2739491
Anti-human TCR γδ; FITC; B1; BD Biosciences; 559878; AB_397353
Anti-human TCR Vα7.2; PE-Cy7; 3C10; BioLegend; 351712; AB_2561994
Anti-human TCR Vα24-Jα18; FITC; 6B11; BioLegend; 342906; AB_1731856
Anti-human TCR Vδ2; FITC; B6; BioLegend; 331406; AB_1089230
Anti-human TNF; BV605; MAb11; BioLegend; 502909; AB_315261

Validation

All antibodies are commercially available and validation statements can be found on the manufacturers' websites using the catalogue number or in the Antibody Registry database (https://antibodyregistry.org) using the RRID. Antibodies were titrated to achieve optimal separation between negative and positive populations.

Flow Cytometry

Plots

Confirm that:

☒ The axis labels state the marker and fluorochrome used (e.g. CD4-FITC).

☒ The axis scales are clearly visible. Include numbers along axes only for bottom left plot of group (a 'group' is an analysis of identical markers).

☒ All plots are contour plots with outliers or pseudocolor plots.

☒ A numerical value for number of cells or percentage (with statistics) is provided.

Methodology

Sample preparation

Liver tissue collection and processing (Exp 1 and 2):
Liver tissue (n=7) and matched blood (n=6) were obtained from patients undergoing liver resection at the Churchill Hospital, Oxford, UK and the University Hospital Basel, Basel, Switzerland (Supplementary Table 1). Patients had no chronic liver disease, active excess alcohol consumption (> 14 g/day), infection, immunosuppression or family history of liver disease.

Disease-free liver tissue was collected from the resection margin, cut into small pieces with a scalpel, and ground through a 70 µm cell strainer. Cells were washed with R10 (RPMI-1640 [Sigma-Aldrich], 10% FBS [Sigma-Aldrich], 1% penicillin-streptomycin [Thermo Fisher Scientific]; 931g, 10 min, 4 °C) and mononuclear cells isolated by density gradient centrifugation on a discontinuous 35%/70% Percoll (GE Healthcare) gradient (931g, 20 min, 21 °C, no brake). Mononuclear cells were collected from the interface and washed with R10 (596g, 10 min, 4 °C). Residual red blood cells were lysed with ACK for 3-5 min. Cells were washed twice (596g, 10 min, 4 °C) and cryopreserved (90% FBS, 10% DMSO [Sigma-Aldrich]) in liquid nitrogen.

Ethics statement:
Samples were obtained with written informed consent through the Oxford Gastrointestinal Illnesses Biobank (REC ref. 16/YH/0247) or under Ethikkommission Nordwest- und Zentralschweiz (EKNZ) numbers EKNZ-2014-362, EKNZ-2016-01188 and EKNZ-2019-02118.

Peripheral blood mononuclear cell (PBMC) isolation:
PBMCs were isolated from fresh whole blood by density gradient centrifugation (Lymphoprep, Axis-Shield) at 931g for 30 min with no brake. Cells were cryopreserved in liquid nitrogen and thawed in complete medium (R10, 1X nonessential amino acids [Thermo Fisher Scientific], 1 mM sodium pyruvate [Thermo Fisher Scientific], 10 mM HEPES [pH 7.0-7.5; Thermo Fisher Scientific], 50 µM β-mercaptoethanol [Thermo Fisher Scientific]) on the day of use.

Stimulation of isolated CD8+/CD3+ T cells for scRNA-seq and scTCR-seq (Exp 3 and 4) or activation marker/cytokine validation:
Pierce streptavidin-coated high-capacity flat-bottom 96-well plates (Thermo Fisher Scientific) were coated with 50 µl biotinylated MR1/5-OP-RU monomer (NIH Tetramer Core Facility) at 10 µg/ml in PBS (Sigma-Aldrich) overnight at 4 °C. Cryopreserved PBMCs were thawed in complete medium. CD8+ T cells were isolated using CD8 MicroBeads (Exp 3; Miltenyi Biotec) and CD3+ T cells using the REAlease CD3 MicroBead Kit (Exp 4 and validation experiments; Miltenyi Biotec) following the manufacturer’s instructions. Isolated CD8+/CD3+ T cells were washed in complete medium and resuspended at 1 × 107 cells/ml. One million (20 h stimulation) or 500,000 (68 h stimulation) cells were added per well to the appropriate 96-well plates (MR1/5-OP-RU-coated plate for TCR and TCR+cytokine stimulation, round-bottom plate for unstimulated and cytokine stimulation). IL-12 (50 ng/ml; R&D Systems) and IL-18 (50 ng/ml; R&D Systems) were added for cytokine stimulation; αCD28 (1 µg/ml; clone: CD28.2; BioLegend) for TCR stimulation; IL-12, IL-18, and αCD28 for TCR+cytokine stimulation; and complete medium for unstimulated cells (final volume 200 µl/well). Cells were incubated for 20 h or 68 h at 37 °C, 5% CO2. For intracellular cytokine staining, brefeldin A (BioLegend) and monensin (BioLegend) were added for the final 4 h.

Tetramer staining (Exp 1 and 2):
Biotinylated human MR1/5-OP-RU and MR1/6-FP monomers were provided by the NIH Tetramer Core Facility. Tetramers were generated using streptavidin-PE (high concentration) or streptavidin-BV421 (both BioLegend) following the NIH Tetramer Core Facility protocol. Tetramer staining was performed for 40 min at 21 °C in FACS buffer (PBS, 0.5% BSA [Sigma-Aldrich], 1 mM EDTA [Sigma-Aldrich]).

Surface staining and cell sorting for scRNA-seq and scTCR-seq (Exp 1-4):
TotalSeq-C hashtag antibodies (BioLegend) were used in Exp 2 and 4. Hashtag antibody dilutions were prepared according to the manufacturer’s instructions. Namely, antibody vials were centrifuged at 10,000g, 30 s, 4 °C, before antibody dilution in

FACS buffer. Diluted hashtags were centrifuged at 14,000g, 10 min, 4 °C. Cells were incubated in Human TruStain FcX (BioLegend) for 10 min at 4 °C before the addition of diluted hashtag antibodies (0.2 µg/well) for 10 min at 4 °C. Surface fluorochrome-conjugated antibodies were added without washing off the hashtag antibodies. Surface staining was performed in Brilliant Stain Buffer Plus (BD Biosciences) for 30 min at 4 °C. Cells were washed twice in PBS with 0.5% BSA, resuspended in presort buffer (PBS, 1% BSA, 25 mM HEPES) containing 3-5 nM SYTOX Green Nucleic Acid Stain (Thermo Fisher Scientific) and incubated for 20 min at 4 °C. Cells were sorted on a BD FACSAria III with an 85 µm nozzle. Sorted cells were collected in RPMI-1640, 10% FBS, 25 mM HEPES, or HBSS (Thermo Fisher Scientific), 50% FBS, 25 mM HEPES. Sort purity was > 99%. For Exp 2 and 4, sorted cells were stained with the TotalSeq-C Human Universal Cocktail V1.0 (BioLegend) according to the manufacturer's instructions. Staining reagents are listed in Supplementary Table 12.

Stimulation of CD56- and CD56+ MAIT cells:
CD3+ T cells were isolated using the REAlease CD3 MicroBead Kit following the manufacturer's instructions. Surface antibody and live/dead (SYTOX Green Nuclear Acid Stain) staining were performed as above, then CD56- and CD56+ MAIT cells (Vα7.2 +CD161hi) sorted on a BD FACSAria III with an 85 µm nozzle. Sorted cells were collected in HBSS, 50% FBS, 25 mM HEPES, then centrifuged at 400g, 5 min, 21 °C and incubated overnight at 37 °C, 5% CO2. Rested cells were washed in complete medium, plated in a 96-well round-bottom plate and stimulated with IL-12 (50 ng/ml) and IL-18 (50 ng/ml) at 37 °C, 5% CO2 for 20 h, with the addition of brefeldin A and monensin for the final 4 h.

Surface marker and intracellular cytokine staining for flow cytometry:
Surface staining was performed in Brilliant Stain Buffer Plus for 30 min at 4 °C. Stained cells were washed twice in FACS buffer. For intracellular cytokine staining, cells were fixed in Cytofix/Cytoperm (BD Biosciences) for 20 min at 4 °C, then washed twice in 1X Perm/Wash (BD Biosciences). Intracellular staining was performed in 1X Perm/Wash for 30 min at 4 °C. Cells were acquired on a BD LSR II flow cytometer with BD FACSDiva Software (v8.0.1). Staining reagents are listed in Supplementary Table 12.

Instrument

Cells sorting was performed on a BD FACSAria III with an 85 µm nozzle. Cells were analysed on a BD LSR II.

Software

FACSDiva (v8.0.1; BD Biosciences) for data collection, FlowJo (v10.8.1; BD Biosciences) for data analysis.

Cell population abundance

Postsort purity, determined for representative samples, was > 99%.

Gating strategy

See Supplementary Figures 1-3.

Exp 1 and 2 - FSC-A vs. SSC-A (lymphocytes), CD45 vs. SSC-A (hematopoietic), FSC-H vs. FSC-A (singlets), CD3ε vs. Dump (live CD3+ T cells; Dump = CD14, CD19, TCR γδ, TCR Vα24-Jα18, TCR Vδ2, SYTOX Green Nucleic Acid Stain). MAIT cells are MR1/5-OP-RU+ and Tmem cells are MR1/5-OP-RU-CCR7-.

Exp 3 - FSC-A vs. SSC-A (lymphocytes), CD3ε vs. Dump (live CD3+ T cells; Dump = CD14, CD19, TCR γδ, TCR Vα24-Jα18, TCR Vδ2, SYTOX Green Nucleic Acid Stain), FSC-H vs. FSC-A (singlets), CD4 vs. CD8α (CD8+ T cells). MAIT cells are CD26 +CD161hiVα7.2+.

Exp 4 - FSC-A vs. SSC-A (lymphocytes), CD3ε vs. Dump (live CD3+ T cells; Dump = CD14, CD19, TCR γδ, TCR Vα24-Jα18, TCR Vδ2, SYTOX Green Nucleic Acid Stain), FSC-H vs. FSC-A (singlets). MAIT cells are CD26+CD161hiVα7.2+.

CD56- and CD56+ MAIT cell sorting and poststimulation analysis:
Sorting - FSC-A vs. SSC-A (lymphocytes), CD3ε vs. Dump (live CD3+ T cells; Dump = CD14, CD19, TCR γδ, TCR Vα24-Jα18, TCR Vδ2, SYTOX Green Nucleic Acid Stain), FSC-H vs. FSC-A (singlets). CD56- and CD56+ MAIT cells are Vα7.2+CD161hiCD56- and Vα7.2+CD161hiCD56+, respectively.
Poststimulation analysis - FSC-H vs. FSC-A (singlets), FSC-A vs. SSC-A (lymphocytes), CD3ε vs. live/dead (live CD3+ T cells). CD56- and CD56+ MAIT cells are Vα7.2+CD161hiCD56- and Vα7.2+CD161hiCD56+, respectively.

Stimulation and activation marker staining - FSC-H vs. FSC-A (singlets), FSC-A vs. SSC-A (lymphocytes), CD3ε vs. Dump (live CD3+ T cells; Dump = CD14, CD19, TCR γδ, TCR Vα24-Jα18, TCR Vδ2, SYTOX Green Nucleic Acid Stain). MAIT cells are CD26 +CD161hiVα7.2+.

Stimulation and cytokine staining - FSC-H vs. FSC-A (singlets), FSC-A vs. SSC-A (lymphocytes), CD3ε vs. live/dead (live CD3+ T cells). MAIT cells are Vα7.2+CD161hi.

ATAC-seq - FSC-A vs. SSC-A (lymphocytes), FSC-A vs. Dump (non-monocyte, non-B, live; Dump = CD14, CD19, live/dead), FSC-H vs. FSC-A (singlets), CD3ε vs. TCR γδ (non-γδ T cells), CD4 vs. CD8α (CD8+ T cells). Naive T cells are CD45RO-CCR7+, MAIT cells are CCR7-MR1/5-OP-RU+ and Tmem cells are CCR7-MR1/5-OP-RU-.

☒ Tick this box to confirm that a figure exemplifying the gating strategy is provided in the Supplementary Information.
