# Supplementary material for: Single-cell analysis of human MAIT cell transcriptional, functional and clonal diversity
Source: Nat Immunol. 2023 Aug 14;24(9):1565–78. doi: 10.1038/s41590-023-01575-1 (PMC10457204; doi:10.1038/s41590-023-01575-1)
Supplement: Supplementary file 4 — Characteristics of liver tissue donors. [file 41590_2023_1575_MOESM4_ESM.pdf]

Donor	Age	Sex	Diagnosis/liver surgery	Background liver	Potential immunomodulatory therapy
1.1	50	F	Resection of primary hepatocellular carcinoma	Minimal steatosis, no fibrosis	Nil
1.2	51	F	R hepatectomy for hemangioma	Normal	Nil
1.3	65	M	R hepatectomy for rectosigmoid tumor metastasis	Normal	Previous chemotherapy (8 cycles > 2 months previously)
1.4	65	M	Resection of primary hepatocellular carcinoma	Mild steatosis, moderate fibrosis	Nil
2.1	37	F	Resection of benign liver adenoma	Normal	Nil
2.2	27	F	Resection of benign focal nodular hyperplasia (FNH)	Mild nodular regenerative hyperplasia, otherwise normal	Nil
2.3	42	F	Resection for benign polycystic liver disease	No steatosis, mild portal fibrosis	Nil

Supplementary Table 1. Characteristics of liver tissue donors. Two patients had primary hepatocellular carcinoma, one had an isolated metastasis from a colonic tumor (this patient had had prior chemotherapy), and the others had benign lesions.
